# Metal ion cofactors modulate integral enzyme activity by varying differential membrane curvature stress†

**DOI:** 10.1039/d4lf00309h

**Published:** 2024-10-25

**Authors:** Paulina Piller, Paul Reiterer, Enrico F. Semeraro, Georg Pabst

**Affiliations:** a Biophysics, Institute of Molecular Biosciences, University of Graz NAWI Graz Graz Austria georg.pabst@uni-graz.at +43 316 380 4989; b BioTechMed Graz Graz Austria; c Field of Excellence BioHealth Graz Austria

## Abstract

Metal ions are well-known cofactors of protein function and stability. In the case of the integral membrane enzyme OmpLA (outer membrane phospholipase A) the active dimer is stabilized by calcium ions. We studied the lipid hydrolysis kinetics of OmpLA in charge-neutral and charged membranes with symmetric or asymmetric transbilayer lipid distributions. In charge-neutral membranes, OmpLA was more active in symmetric bilayers due to the lower differential curvature stress between membrane leaflets. Strikingly, this behavior was completely reversed in charged bilayers. Measurements revealed intrinsic molecular shape changes in the charged lipids upon addition of calcium. This effectively reduces the differential curvature stress in charged asymmetric membranes leading to increased protein activity. This conclusion is further supported by similar effects observed upon the addition of sodium ions, which also alter the shape of the lipids, but do not specifically interact with the protein. Additional lipid–protein interactions likely contribute to this phenomenon. Our findings demonstrate that ion cofactors not only interact directly with membrane proteins but also modulate protein activity indirectly by altering the effective molecular shape of charged lipid species.

## Abstract 2

Metal ion cofactors not only stabilize the conformation of membrane proteins but also modulate their function by inducing ion-mediated changes in the intrinsic curvature of the surrounding lipids.

## Graphical abstract

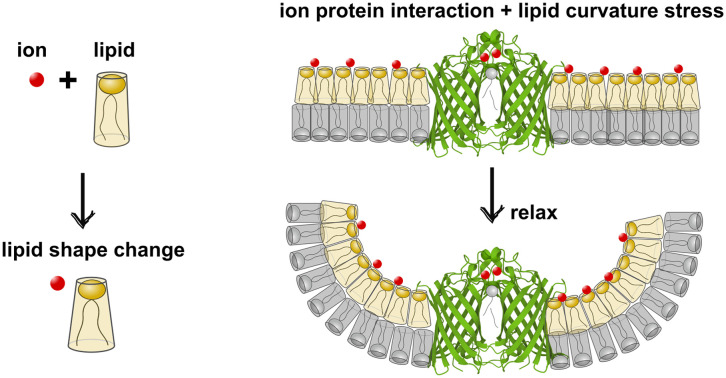

## 1 Introduction

Membrane proteins constitute about one-third of the human proteome^1,2^ and have fundamentally different functions compared to water-soluble proteins. Membranes provide the environment for embedded proteins and facilitate their functions through a complex interplay of specific and non-specific interactions.^3^ This dynamic enables essential roles such as transmembrane signaling, transport, and catalysis. Fundamental understanding of the underlying physicochemical principles thus holds key to diverse applications in drug development^4^ and biotechnology.^5^ Recently, the impact of an asymmetric transbilayer distribution of lipids—a hallmark of all plasma membranes—on membrane protein function has garnered significant scientific interest.^6^

In this Communication, we revisit the effect of membrane asymmetry on the outer membrane phospholipase A (OmpLA). OmpLA is a well-characterized membrane enzyme with an antiparallel β-barrel fold, which hydrolyzes phospholipids upon dimerization in both native and artificial membranes.^7^ Previously we demonstrated that OmpLA activity could be allosterically modulated by differential lateral curvature stress emerging from differently composed membrane leaflets.^8^ Specifically, we observed a slowing down of the hydrolysis kinetics in bilayers composed of the charge-neutral lipids 1-palmitoyl-2-oleoyl-*sn-glycero*-3-phosphocholine (POPC) and 1-palmitoyl-2-oleoyl-*sn-glycero*-3-phosphoethanolamine (POPE) with increasing compositional asymmetry between the two membrane leaflets.

Here we interrogate the effect of the anionic lipid 1-palmitoyl-2-oleoyl-*sn-glycero*-3-phosphoglycerol (POPG) on OmpLA activity with a specific focus on the role of the Ca^2+^ cofactor. Cofactor ions are generally important for protein folding, regulation and stability.^9,10^ In the case of OmpLA, Ca^2+^ ions are considered to stabilize active dimers by mutually aligning the active sites located on the outer protein surface.^7^ On the other hand, Ca^2+^ ions are also known to strongly bind and affect the structural properties of membranes containing anionic lipids.^11,12^ We thus hypothesized that asymmetrically distributed charged lipids might lead to distinct behavior of OmpLA upon activation with Ca^2+^ through a mechanical coupling to collective membrane properties.

## 2 Results and discussion

We expressed, purified, and reconstituted OmpLA lacking the signal sequence into liposomes of approximately 100 nm size, as detailed previously;^8^ see also the ESI† for details regarding materials and methods. Two types of proteoliposomes were prepared: asymmetric and symmetric with respect to the transbilayer lipid distribution. Asymmetric proteoliposomes were created by exchanging the outer leaflet of OmpLA proteoliposomes using methyl-β-cyclodextrin (mβCD). Symmetric proteoliposomes were prepared by reconstituting OmpLA into vesicles of the same lipid mixtures of the asymmetric systems, which was determined from high-performance thin layer chromatography (HPTLC). Table 1 provides an overview of the lipid composition of all studied samples. All experiments were carried out at 35 °C.

**Table 1:** Overview of initial lipid composition of the reported symmetric and asymmetric proteoliposomes. The hydrocarbon chains of all phospholipids are identical (*sn*-1: palmitoyl; *sn*-2: oleoyl). The first column gives the overall molar ratio for both symmetric and asymmetric vesicles. The second and third columns detail the inner leaflet and the outer leaflet compositions. Subscripts report the fraction of the overall lipid composition assuming that no lipids flip to the inner leaflets during exchange

Sample	Molecular ratio (mol : mol)	Inner leaflet fraction	Outer leaflet fraction
PE/PC_1_^a^	34 : 66	PC_0.5_	PE_0.34_; PC_0.16_
PE/PC_2_^a^	47 : 53	PE_0.19_; PC_0.31_	PE_0.35_; PC_0.15_
PE/PC/PG	25 : 37 : 38	PC_0.25_; PG_0.25_	PE_0.25_; PC_0.12_; PG_0.13_
PE/PG	37 : 63	PG_0.5_	PE_0.37_; PG_0.13_

a From ref. 8.

**Fig. 1:**
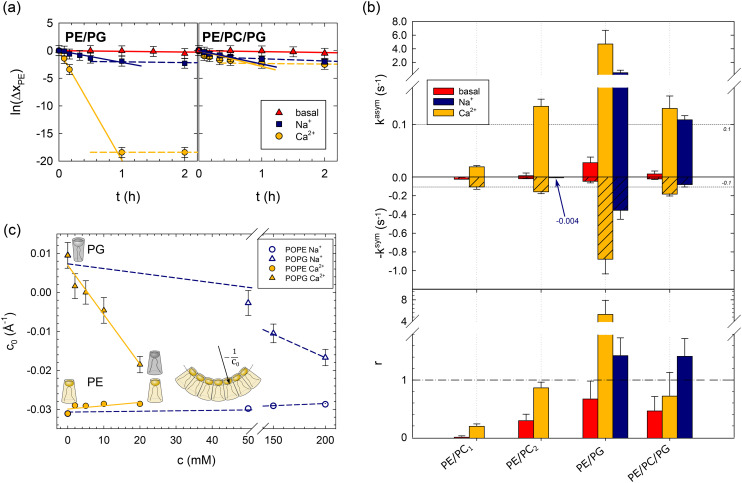
Effects of lipid asymmetry on the activity of OmpLA in plain buffer (basal) and upon the addition of 200 mM NaCl, or 20 mM CaCl_2_. Panel (a): Analysis of POPE hydrolysis kinetics of asymmetric PE/PG and PE/PC/PG proteoliposomes; see Table 1 for composition. Solid lines show fits according to eqn (1), and dashed lines indicate deviations from the initial hydrolysis kinetics. Panel (b): Normalized hydrolysis rates in asymmetric (top) and symmetric (middle) proteoliposomes, as well as the ratio *r* = *k*^asym^/*k*^sym^ (bottom). Note the different ordinate scales for *k*^asym^ and *k*^sym^; the horizontal dashed line at |*k*^asym^| = |*k*^sym^| = 0.1 s^−1^ allows a tentative comparison. *k*^sym^ was multiplied by −1 for data presentation reasons. The blue arrow and number for PE/PC_2_ indicate the observed hydrolysis rate upon adding of Na^+^ ions. Panel (c): Intrinsic lipid curvatures of POPE and POPG as a function of salt concentration. The insert shows the definition of *c*_0_, for details see ref. 14.

Time-resolved HPTLC was used to determine the kinetics of lipid hydrolysis upon adding 20 mM CaCl_2_, a salt concentration that is way above physiological conditions, but consistent with previous OmpLA activity studies.^8,13^ Using this salt concentration thus provides a common reference point. Focusing on POPE, the only lipid common to all studied samples, the solution of the corresponding rate equation^8^ is expressed as:

1 ln(Δ*x*_PE_) = −*k*_PE_*t*,

where *k*_PE_ represents the lipid hydrolysis rate, *t* is time and Δ*x*_PE_ = (*x*_PE_(*t*) − *x*^∞^_PE_)/*x*^0^_PE_ − *x*^∞^_PE_. Here *x*^0^_PE_ and *x*^∞^_PE_ are the molar fractions of POPE at the start and at the end of the hydrolysis process, respectively. This linearized form of the rate equation facilitates a straightforward linear regression to determine *k*_PE_'s. Factors such as overall changes in lipid composition or lipid flip-flop are expected to influence hydrolysis kinetics as the experiment progresses. Therefore, only the initial changes in POPE content, following the given rate equation, were considered (Fig. 1a).

Fig. 1b summarizes the lipid hydrolysis results. The rates *k*^asym^ and *k*^sym^ were normalized by the overall lipid concentration and protein copy number (Table S2†), as detailed previously.^8^ The top panel presents *k*^asym^, including previously reported data for charge-neutral POPE/POPC mixtures (PE/PC_1_, PE/PC_2_). The increase in *k*^asym^ from PE/PC_1_ to PE/PC_2_ can be explained by the less asymmetric distribution of POPE in PE/PC_2_ (Table 1), which leads to lower curvature stress differences between the two leaflets.^8^ Turning to the here studied charged bilayers, the approximately 35 times higher *k*^asym^ in PE/PG is highly remarkable. Also the basal activity of the enzyme was about 6 times higher in this system.

The middle panel of Fig. 1b shows the normalized POPE hydrolysis rates in the same but symmetric lipid mixtures. Note the different scale compared to the upper panel and that *k*^sym^ was multiplied by −1 for presentation purposes. The activity of OmpLA was within experimental uncertainty about equal in the two charge-neutral lipid mixtures.^8^ Again, the hydrolysis rate in the symmetric POPE/POPG mixture increased, also in the absence of Ca^2+^, but to a much lesser extent than in the case of asymmetric proteoliposomes.

It is most instructive to compare the activity of OmpLA in symmetric and asymmetric proteoliposomes for a given lipid mixture using the ratio of the normalized hydrolysis rates *r* = *k*^asym^/*k*^sym^ (Fig. 1b, bottom panel). For the charge-neutral POPE/POPC lipid mixtures, *r* < 1, indicating that the hydrolysis proceeds faster when the lipids are symmetrically distributed.^8^ We observed a markedly different behavior in PE/PG: *r* > 1 upon the addition of Ca^2+^ ions, whereas *r* < 1 in the absence of Ca^2+^ ions. This means that hydrolysis proceeds faster in symmetric than in asymmetric POPE/POPG mixtures under basal conditions, while the addition of Ca^2+^ accelerates hydrolysis more in the asymmetric than in the symmetric mixture. This clearly suggests that effects beyond the classical cofactor role of Ca^2+^ are at play.

Encouraged by our previous success in explaining OmpLA activity in charge-neutral asymmetric and symmetric bilayers through membrane-mediated allostery,^8^ we hypothesized that ion-mediated effects on lipid properties could feedback to influence protein function. Specifically, the intrinsic lipid curvature (*c*_0_), which relates to the apparent lipid molecular shape or membrane curvature stress,^15,16^ is one of the most sensitive parameters of our allosteric model. For instance, *c*_0_ = −0.032 Å^−1^ for POPE at 35 °C in water,^14^ indicating an inverted cone-like shape. Notably, calcium-mediated changes in *c*_0_ of the two anionic lipids, cardiolipin and phosphatidic acid, have been reported and attributed to modulation of inter-headgroup repulsion.^17^ The screening of electrostatic headgroup interactions, combined with calcium's potential to bind to two headgroups simultaneously,^11^ reduces the lipid's apparent headgroup size, leading to an effective shift toward inverted cone-like molecular shapes.^17^ We hypothesized that Ca^2+^ might modulate *c*_0_ of POPG through a similar mechanism.

To test this hypothesis, we measured the intrinsic lipid curvatures of POPE and POPG in salt solutions using small-angle X-ray scattering (SAXS), as established previously;^14,18^ see the ESI.† Consistent with previous measurements on dioleoyl phosphatidylethanolamine,^17^ we found that Ca^2+^ barely affected the *c*_0_ of POPE. In turn, the intrinsic lipid curvature of POPG decreased significantly in the same Ca^2+^ concentration range from slightly positive to negative *c*_0_ values (Fig. 1c). Thus, Ca^2+^ renders the apparent molecular shape of POPG more similar to POPE's inverted conical form. This leads to changes in the curvature stress distributions in the membrane that depend on the lipid composition of each leaflet that could indeed affect OmpLA activity.

We conducted several control experiments to corroborate the notion that ion-mediated effects may couple mechanically through lipids to influence OmpLA activity. First, we reduced the overall surface charge of the lipid bilayer by preparing proteoliposomes with equimolar mixtures of POPG and POPC in each leaflet, as well as POPE, most of which was located in the outer leaflet in the asymmetric lipid systems (see Table 1). Both hydrolysis rates (*k*^asym^, and *k*^sym^) were significantly reduced, and *r* < 1 (Fig. 1b). This is consistent with a reduced effect of Ca^2+^ on the average *c*_0_ of the POPC/POPG mixture, assuming negligible effects of ions on the intrinsic curvature of POPC.

Next, we added just Na^+^, an ion not considered a cofactor of OmpLA. The rationale for these controls was that if lipid curvature effects activate OmpLA, other ions affecting *c*_0_ should yield similar protein activities. Our SAXS experiments showed that Na^+^ can also change the shape of POPG to an inverted conical form, albeit requiring higher concentrations. POPG *c*_0_ values comparable to those with 20 mM CaCl_2_ occur at 200 mM NaCl (Fig. 1c).

We thus added 200 mM Na^+^ to our PE/PG and PE/PC/PG samples and observed that these ions can indeed activate OmpLA in both symmetric and asymmetric proteoliposomes, although with somewhat reduced hydrolysis rates (Fig. 1b). Moreover, consistent with our results upon adding Ca^2+^, the hydrolysis was faster in asymmetric PE/PG samples (*r* > 1). Only in the case of PE/PC/PG did we find *r* < 1 in the presence of Ca^2+^, while *r* > 1 when adding Na^+^. These different *r*-values, along with the slightly lower *k*^asym^ and *k*^sym^ values in the presence of Na^+^, suggest that Ca^2+^ still acts as cofactor for OmpLA activity in charged bilayers in its classical sense.

Finally, we added 200 mM Na^+^ to symmetric PE/PC_2_ proteoliposomes (see Table 1), which are charge-neutral samples. If changes in the intrinsic curvature of POPG are required to activate the enzyme, we would expect no effect from Na^+^ in PE/PC_2_. Consistently, we observed *k*^sym^ = (4 ± 3) × 10^−3^ s^−1^, which is within the range of the basal protein activity (see arrow in Fig. 1b).

We also explored whether the observed effects could be quantitatively rationalized within our previously reported allosteric model.^8^ Briefly, this model considers the work associated with activating OmpLA dimers against curvature stress differences between the membrane leaflets. Comparing symmetric and asymmetric membranes, this leads to:2
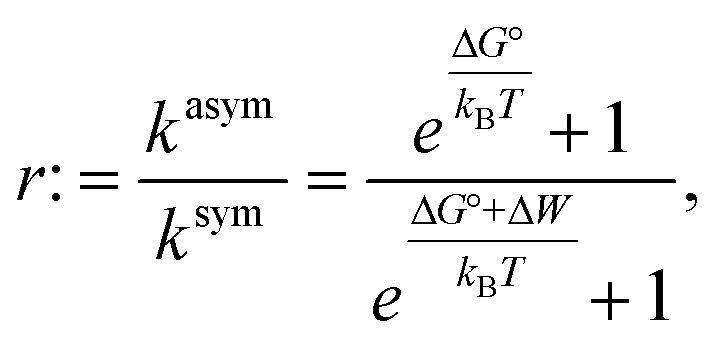
where Δ*G*° is the free energy of activation of the enzyme in symmetric bilayers, *k*_B_ is Boltzmann's constant, *T* is the absolute temperature, and Δ*W* = Δ*W*^sym^ − Δ*W*^asym^ is the difference of work for protein activation in symmetric and asymmetric bilayers. Δ*W* can be estimated based on the protein shape and the lipid composition using intrinsic lipid curvature and bending rigidity data.^8^ This leaves Δ*G*° as the only adjustable parameter.

**Fig. 2:**
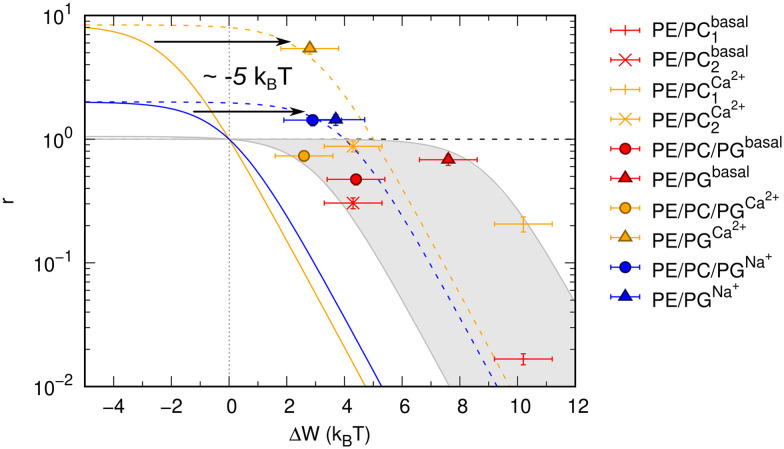
Allosteric modulation of OmpLA activity by curvature stress differences between symmetric and asymmetric membranes. The grey area includes a range of possible Δ*G*° values of −9 to −3*k*_B_*T* (according to eqn (3)). Colored solid and dashed lines show how a shift of values by about −5*k*_B_*T* (possibly additional electrostatic contribution) could represent the data for *r* > 1.

Results shown in Fig. 2 demonstrate a good overall agreement of all samples with Δ*G*° ranging between −9 to −3*k*_B_*T*. Only the three samples for which we found *r* > 1 do not conform to the applied model. According to this model *r* values significantly larger than 1 can only occur for negative Δ*W* values and when Δ*G*° ≳ −1*k*_B_*T* because3
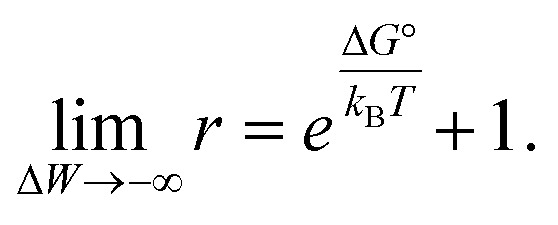
Negative Δ*W* values occur if the work required for OmpLA activation is higher in asymmetric bilayers. Based on the available structural and elastic parameters of the given lipid mixtures (Table S1†), our calculations show that Δ*W* decreases in the presence of Ca^2+^ and Na^+^ for the charged bilayers (but not for the charge-neutral ones), but never reaches negative values (Fig. 2). Furthermore, Δ*G*° ≳ −1*k*_B_*T* implies that the enzyme would be barely active in symmetric membranes, as the free energy of activation would be on the order of thermal energy (unstable OmpLA dimers) or even positive (energetically unfavorable). This clearly does not align with our findings (Fig. 1b, mid-panel).

We speculate that additional electrostatic interactions between POPG and OmpLA might further modulate protein activity, resulting in *r* > 1. This could also explain the increased basal activity of the enzyme in bilayers containing POPG. Our estimations indicate that such contributions would need to shift the free energies by approximately 5*k*_B_*T* to ‘recover’ the allosteric model (Fig. 2). Currently, we lack any clear evidence. However, it is noteworthy that Wu *et al.*^19^ reported interactions between the extracellular loops of OmpLA and lipid A—a much more complex, but also anionic, lipid.

Finally, one might question whether the orientation of OmpLA within the bilayers plays any role. Since this factor was not controlled during the reconstitution process, we assume a random distribution of OmpLA, with both inside-out and outside-in orientations. Although these populations may exhibit significantly different initial hydrolysis rates, we expect ions to quickly access the vesicle lumen once the enzymes begin hydrolyzing the phospholipids. This should harmonize these differences at large. Given that the time resolution of our experiment is on the scale of minutes—too slow to detect such rapid processes—we think that the orientation of OmpLA should not significantly affect the present results.

## 3 Conclusions

**Fig. 3:**
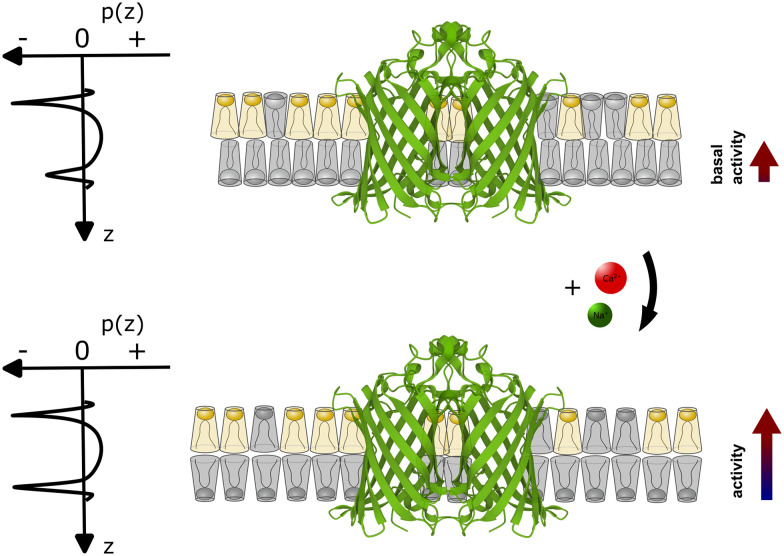
Schematic of coupling between the ion-mediated change of POPG shape (grey lipid) in an asymmetric membrane containing an OmpLA dimer (Protein Data Bank code: 1QD6 (ref. 21)). The upper scheme depicts the system at basal conditions. POPG has a weakly expressed cone-like shape (*c*_0_ ≳ 0), while POPE (yellow lipid) adopts an inverted conical shape (*c*_0_ < 0). Imposing a packing into flat monolayer creates significant curvature stress in the POPE enriched leaflet, but not in the POPG-rich leaflet. This leads to differential curvature stress between the two leaflets as indicated by the asymmetric lateral pressure profile *p*(*z*). With POPG adopting a more inverted cone-like shape in the presence of Ca^2+^ or Na^+^ (lower panel) the curvature stress in the POPG-rich leaflet also increases. However, the differential lateral stress between the membrane leaflets is now partially alleviated (*p*(*z*) becomes more symmetric), leading to an increase of enzyme activity.

Our experimental evidence suggests that the role of Ca^2+^ ions as cofactors for OmpLA activity needs to be reevaluated in the context of charged membranes, considering that native membranes are typically charged. While Ca^2+^ clearly assists in stabilizing active OmpLA dimers, the ion-mediated change in the membrane curvature stress contribution of POPG also strongly modulates enzyme activity for the presently studied samples; see Fig. 3 for schematic. Although Na^+^ ions primarily screen electrostatic repulsion between the charged PG headgroups,^20^ while Ca^2+^ strongly binds to PG,^12^ they can induce similar OmpLA activities by altering the intrinsic lipid curvature.

Despite the need for further experimental or theoretical refinements to achieve quantitative agreement with an allosteric model, our findings reveal a remarkable layer of functional complexity in membranes: ions can modulate the activity of an integral membrane protein by modulating lipid curvatures. Fundamentally, our results suggest that active or passive transport of ions (including Na^+^ and Ca^2+^) through cellular membranes,^10^ creates a time signature of differential membrane curvature stress that could contribute to protein function. On an applied level, such effects could be harnessed to potentiate or attenuate the activity of specific proteins in sensing devices. The significance of this mechanism, compared to specific modulators of protein function such as ligands, remains to be demonstrated.

## Data availability

All HPTLC data are available from https://doi.org/10.5281/zenodo.13305374 and all SAXS data can be downloaded from https://doi.org/10.5281/zenodo.13300234.

## Author contributions

P. P. performed and analyzed HPTLC experiments, P. R. performed and analyzed SAXS experiments. E. S. and G. P. analyzed rate equations and wrote the paper.

## Conflicts of interest

There are no conflicts to declare.

## Supplementary Material

LF-002-D4LF00309H-s001
