# Supplementary material for: Metal ion cofactors modulate integral enzyme activity by varying differential membrane curvature stress
Source: RSC Appl Interfaces. 2024 Oct 25;2(1):69–73. doi: 10.1039/d4lf00309h (PMC11514723; doi:10.1039/d4lf00309h)
Supplement: LF-002-D4LF00309H-s001 [file LF-002-D4LF00309H-s001.pdf]

Supplementary Information (SI) for RSC Applied Interfaces.
This journal is © The Royal Society of Chemistry 2024

Supplementary Information

Metal Ion Cofactors Can Modulate Integral Enzyme Activity By Varying Differential Membrane Curvature Stress

Paulina Piller, Paul Reiterer, Enrico F. Semeraro, and Georg Pabst

1 Materials

All lipids (POPE, POPC, POPG) were purchased from Avanti Polar Lipids (Alabaster, AL, USA). $CHCl_3$, MeOH, EtOH, tris(hydroxy-methyl) aminomethane (Tris), NaCl, $CaCl_2$, $MgCl_2$, $MnCl_2$, D(+)-saccharose, isopropyl-β-d-thiogalacto-pyranoside (IPTG), 5,5'-dithiobis-(2-nitrobenzoic acid) (DTNB), urea, glycine, peptone, yeast extract, $CaCl_2$, NaCl, H_2SO_4 (96 % v/v), $Na_2S_2O_5$, Na_2SO_3 were obtained from Carl Roth (Karlsruhe, Germany). Methyl-β-cyclodextrin (mβCD) and lauryldimethylamin-N-oxid (LDAO), $HClO_4$ (67−72 % v/v), ammonium heptamolybdate tetrahydrate and *cis*-9-tricosene were acquired from Sigma-Aldrich (Vienna, Austria); ethylendiaminetetraacetatic acid (EDTA), 1-amino-2-hydroxy-naphthalin-4-sulfonic acid (ANSA) and KH_2PO_4 were from Merck (Darmstadt, Germany).

2 Methods

2.1 Protein Production and Purification

OmpLA without signal sequence was expressed in *Escherichia coli* BL21(DE3) cells (Novagen, Merck, Darmstadt, Germany) as inclusion bodies (IBs) utilizing a pET-24a (+) expression vector (Merck, Darmstadt, Germany). After inoculation of the lysogeny broth medium with *E. coli* BL21(DE3) cells, the production of OmpLA was induced by the addition of IPTG (0.4 mM final concentration). Incubation lasted for 3 h at 37 °C under agitation. Cells were harvested, washed and resuspended in 1:10 (wt/vol) ice-cold breakage buffer (50 mM Tris, 40 mM EDTA, 25 % (wt/vol) sucrose, pH 8.0). Then, cells were sonicated with a sonopuls HD 2070 homogenizer (Bandelin, Berlin, Germany) for 10 min (pulse: 30 s, pause: 30 s) at an amplitude of 40 %. The cell lysate was centrifuged for 1 h at 4 °C and 7000 g, the pellet was resuspended in washing buffer (10 mM Tris, 1 mM EDTA, pH 8.0), centrifuged again with the same settings and dissolved in solubilization buffer (20 mM Tris, 2 mM EDTA, 8 M urea, 100 mM glycine) at 4 °C under agitation overnight. Solubilized IBs were centrifuged for 30 min at 4 °C and 7000 g. The protein concentration of the supernatant was determined with a Nanodrop ND-1000 spectrophotometer (Peqlab Biotechnology GmbH, Erlangen, Germany) by measuring the absorbance (A) at 280 nm, and using the reference value at 10 g/l, $A^{1\%}_{\mathrm{OmpLA}} = 26.68$ (calculated from ProtParam, Expasy, the Swiss Bioinformatics Resource Portal). For blank measurement buffer was used.

Refolding of OmpLA was executed by adding the refolding buffer by drop dilution under agitation at 50 °C to reach final concentrations of 0.33 mg/mL OmpLA, 20 mM Tris, 2 mM EDTA, 0.80 M urea, 10 mM glycine and 35 mM LDAO (pH 8.3). The mixture was further agitated for 16 h at 50 °C. The refolded fraction was centrifuged for 15 min at 4 °C and 7000 g and filtered through a 450-nm poly (-vinylidene fluoride) filter (Carl Roth, Karlsruhe, Germany). The folded protein fraction was separated from the unfolded one by anion exchange chromatography on a 20 ml (4x5 ml) tandem HiTrap DEAE column (GE Healthcare, Solingen, Germany) in 20 mM Tris, 2 mM EDTA, 35 mM LDAO (pH 9.5) with a two-step gradient of 105 mM and 1.5 M KCl. The pooled folded fractions were dialyzed [20 mM Tris, 2 mM EDTA, 12 mM LDAO (pH 8.3)] overnight and concentrated on a 5 ml Resource Q column (GE Healthcare, Solingen, Germany) in 20 mM Tris, 2 mM EDTA and 12 mM LDAO (pH 8.3). The protein was eluted with 20 mM Tris, 2 mM EDTA, 12 mM LDAO and 1.5 mM KCl (pH 8.3) and further desalted on a PD-10 column (GE Healthcare, Solingen,

Germany) using 20 mM Tris, 2 mM EDTA and 2 mM LDAO (pH 8.3). The final protein concentration was measured as described above.

2.2 Vesicle Preparation

Lipids (POPC, POPE, POPG) were dispersed in a 2:1 vol/vol chloroform/methanol mixture, dried under a stream of nitrogen and stored in vacuum overnight to ensure complete solvent evaporation. For lipid hydration the reconstitution buffer (20 mM Tris, 2 mM EDTA, pH 8.3) was added. The formation of lipid vesicles was achieved by intermittent vigorous vortexing at 15 °C $> T_m$ (lipid melting temperature) for 1 h. Large Unilamellar Vesicles (LUVs) were prepared by 31-fold extrusion (mini extruder: Avanti Polar Lipids, Alabaster, AL, USA) through a polycarbonate filter (Whatman Nuclepore^{TM} Track-Etched Membranes from Merck, Darmstadt, Germany) with a pore diameter of 100 nm. LUV formation was assisted in the case of POPC by doping the bilayers with 5 mol% POPG. POPG does not affect membrane structure or protein activity at presently low concentrations (see below). Vesicle size was checked with dynamic light scattering (DLS) to be around 100 nm using a Zetasizer Nano ZSP (Malvern Panalytical, Malvern, UK).

2.3 Inverted Hexagonal Phase Preparation

Fully hydrated H_{II} phases were prepared as detailed previously[1]. In brief, stock solutions were mixed at appropriate molar ratios and added to 300 μl ultra-pure water (18 MΩ/cm^2, organic solvent/water ratio = 2.55 vol/vol), which was incubated before mixing at 65°C in 20 ml test tubes. The mixture was then quickly mounted on a modified rapid solvent exchange apparatus[2] to remove the organic solvent within 5 min by adjusting the vacuum pressure to $400 - 500$ mbar, using a vortex speed of 600 rpm and an Ar flow of 60 ml/min. The final samples contained 12 wt.% tricosene.

2.4 Protein Reconstitution

For reconstitution, OmpLA solubilized in 2 mM LDAO was added drop by drop to lipid vesicles at 35 °C and 350 rpm in a thermomixer (Eppendorf, Hamburg, Germany) to reach a final mole ratio of lipids / proteins of 300: 1 (600: 1 and 900: 1, respectively). LDAO was removed by dialysis against 50 mM Tris, 2 mM EDTA and 200 mM NaCl (pH 8.3) overnight. The proteoliposomes were extruded 31 times through a 100 nm polycarbonate filter.

2.5 Preparation of Asymmetric Proteoliposomes

Asymmetric proteoliposomes were prepared following the heavy donor cyclodextrin exchange protocol[3]. Acceptor and donor lipids were weighed (1:2 mol/mol). Acceptor vesicles were proteoliposomes (LUVs) with various lipid compositions. Donor lipids were POPE multilamellar vesicles (MLVs) in 20 wt% sucrose, obtained by 5 freeze/thaw cycles. Donor vesicles were diluted 20 times with water and centrifuged at 20000 g for 30 min. The pellet was suspended in 35 mM mβCD (lipid:mβCD 1:8 mol/mol) and incubated for 2 h at 40 °C under agitation. Proteoliposomes were added and incubated for 30 min. The exchange was stopped by 8-fold dilution with water. The mixture was centrifuged at 20000 g for 30 min. The removal of residual cyclodextrin and sucrose was done by washing the sample three times with H_2O using 15 ml Amicon centrifuge filters (100 kDa cut-off, Merck, Darmstadt, Germany) at 5000 g. Asymmetric vesicles were concentrated to $<$ 500 µl. To ensure the absence of donor MLVs, the vesicle size was checked by DLS.

2.6 Phosphate Assay

The lipid samples were carbonized first in a heated metal block at maximum heat. After cooling, 0.4 ml acid mixture (9:1 vol/vol conc. H_2SO_4:$HClO_4$) was added and the sample was heated up again for 30 min. 9.6 ml of a reaction mixture (22 ml reagent A: 10.5 mM ANSA, 0.7 mM $Na_2S_2O_5$, 40 mM Na_2SO_3; 500 ml reagent B: ammonium heptamolybdate tetrahydrate (0.26 %)) was added to the cool samples. After mixing, the tubes were placed in a sand bath at 90 °C for 20 min. The extinction of the cooled samples was measured on a Spectrophotometer Onda V-10 Plus (Labbox Labware, S.L., Barcelona, Spain) at 830 nm. The phospholipid concentration was calculated by using a calibration curve (3.2 mM KH_2PO_4 as phosphate-standard-solution containing $1 - 14$ μg phosphor).

2.7 High Performance Thin Layer Chromatography (HPTLC)

The enzymatic degradation of the proteoliposomes was determined by TLC. After lipid extraction against organic solvent (2:1 vol/vol chloroform/methanol) based on the Folch extraction method [4], the samples were spotted on a silica plate (Sigma-Aldrich, Steinheim, Germany) with the automatic TLC sampler 4 (CAMAG, Muttenz, Switzerland). The mobile phase in the developing chamber was a solvent mixture composed of 32.5:12.5:2 vol/vol/vol $CHCl_3$/MeOH/H_2O. After drying, the plate was immersed in a developing bath (5.08 g $MnCl_2$ dissolved in 480 ml H_2O, 480 ml EtOH and 32 ml H_2SO_4), which is sensitive to double bonds, and dried for 15 min at 120 °C [5]. To quantify the lipid concentrations the plate was scanned with the TLC scanner 3 (CAMAG, Muttenz, Switzerland) and further analyzed with WinCats software.

2.8 Small-angle X-ray scattering (SAXS)

SAXS experiments were performed using a SAXSpoint5.0 camera (Anton Paar, Graz, Austria) connected to a MetalJet (Excillum, Kista, Sweden) with a liquid, Ga-rich alloy jet anode. This system was equipped with an Eiger R1 M detector (Dectris, Baden- Daettwil, Switzerland). Samples were contained in paste cells (Anton Paar) and exposed to X-rays for 2 minutes (12 frames à 10 s). All samples were equilibrated for 10 minutes at 35°C (TC 150, Anton Paar) for 10 minutes prior to measurement. The sample-to-detector distance was 700 mm. Data were background corrected and integrated using SAXSanalysis (Anton Paar).

3 Data Analysis

3.1 Rate Equations

Time-resolved HPTLC data for phospholipid hydrolysis can be described empirically using the rate equations [6]

$$\begin{aligned} \dot{x_P}(t) &= -(k_1 + k_2)x_P(t) = -k_P x_P(t) \\ \dot{x_L}(t) &= k_1 x_P(t) - k_L x_L(t) \end{aligned} \tag{1}$$

where x_P is the molar fraction of diacyl phospholipids, x_L is the concentration of lyso-lipid (hydrolyzed at *sn-1*), k_1 is the hydrolysis rate of *sn-1* hydrcarbons, k_2 is the hydrolysis rate of *sn-2* hydrcarbons, and k_L is the hydrolysis rate of x_L. Given the boundary conditions for $t = 0$ and $t \to \infty$, the solutions of Equation (1) are

$$\begin{aligned} x_P(t) &= \left(x_P^0 - x_P^\infty\right) e^{-k_P t} + x_P^\infty \\ x_L(t) &= \left(x_L^0 - x_L^\infty\right) e^{-k_L t} + \\ &+ k_1 \frac{x_P^0 - x_P^\infty}{k_L - k_P} \left(e^{-k_P t} - e^{-k_L t}\right) + x_L^\infty, \end{aligned} \tag{2}$$

from which Eq. (1) in the main text follows.

3.2 Instrinsic Lipid Curvatures

SAXS data of H_{II} phases were analyzed as detailed previously [1,7]. Briefly, the intrinsic lipid curvature is derived from finding the monolayer curvature of H_{II} phases at the neutral plane, R_0 (Fig. S1 a). Lipids, which do not form H_{II} phases are mixed with dioleoyl phosphatidylcholine (DOPE), i.e. they constitute guest lipids, within a matrix of host lipids that form a H_{II} phase. In this case the curvature of the guest lipid is derived from

$$c_0^g = \frac{c_0^{\text{mix}}\left[(1-x) + x\xi\right] - c_0^h(1-x)}{x\xi}, \tag{3}$$

where c_0^{mix} is the monolayer curvature of the DOPE/lipid mixture, c_0^h is the intrinsic curvature of DOPE, x is the mole fraction of guest lipid and $\xi = b_g/b_h$ is the ratio of arc lengths of host and guest lipids (Fig. S1 b). Figure S1 c shows selected SAXS patterns of POPE and POPG/DOPE mixtures in the salt solutions. Figure S1 d gives a schematic of different monolayer curvatures and the related apparent lipid shape.

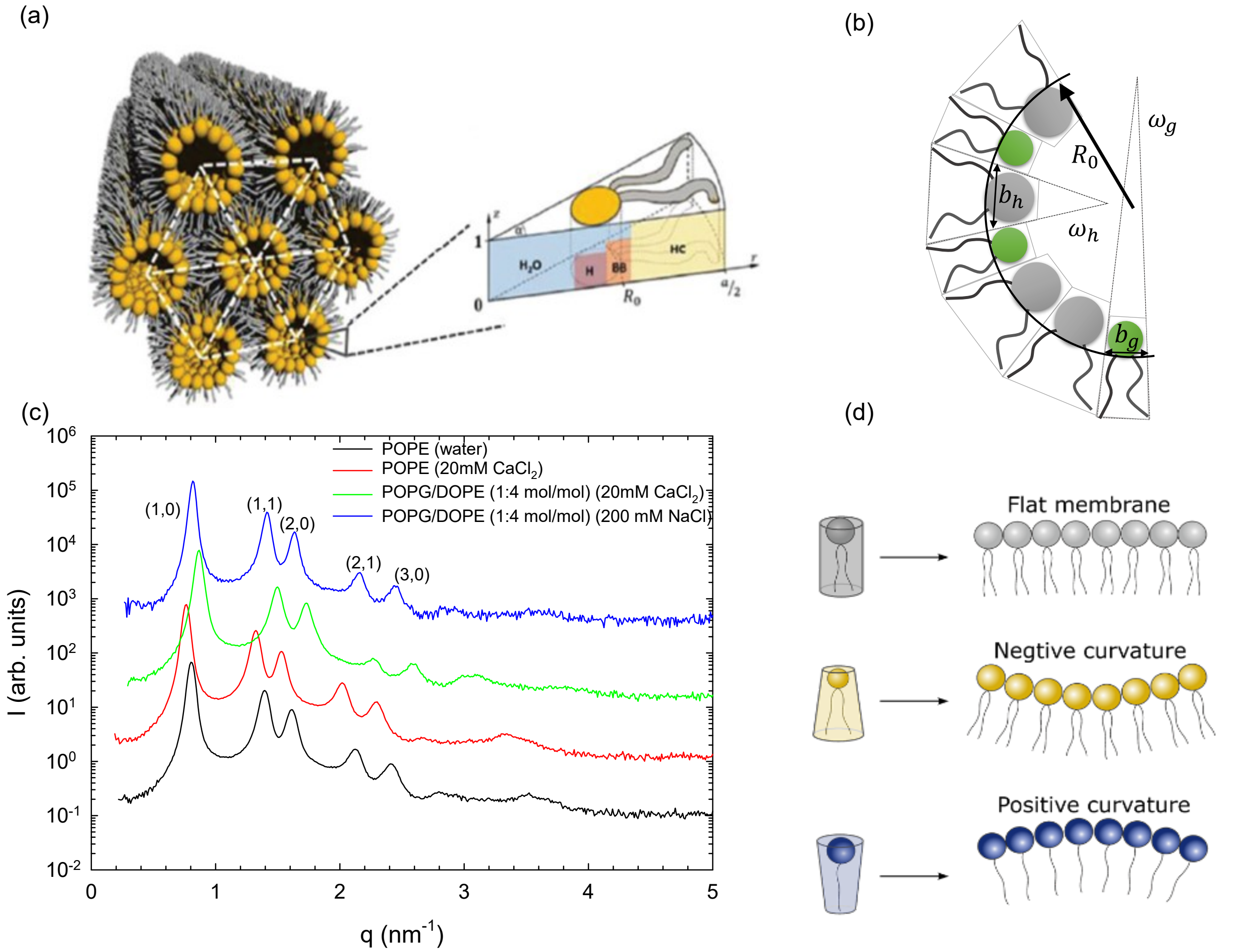

Figure S1: Illustration of H_{II} phase analysis. Panel (a): H_{II} phases are modeled globally by considering different sections of lipid molecules (H…headgroup, BB…backbone, HC…hydrocarbons) as slabs with given electron density. Panel (b): Schematic of the transverse cross-section of a H_{II} phase consisting of two types of wedge-shaped lipids of angles ω_h and ω_g, respectively, and effective in-plane headgroup dimensions b_h and b_g, respectively. The circular arc represents the neutral surface of radius R_0. Panel (c): Selected scattering patterns at 35 °C, including Miller indices. Panel (d): Schematic of lipid monolayer curvatures. Per definition positive curvatures describe surfaces bending toward the water phase and vice versa for negative curved monolayers.

Following our previous reports[1,7], we applied a Bayesian probability theory for optimization of the adjustable parameters. Within this framework any information on the adjustable parameters is represented in probability distribution functions and the model is formulated in terms of a likelihood function, which is integrated using a Markov Chain Monte Carlo (MCMC) algorithm[8].

3.3 Allosteric Model

Briefly, assume that the protein population is divided into predominantly active (A) and inactive (N) states of Ca^{2+}-stabilized dimers. The equilibrium constant between states $A \rightleftharpoons N$ is $K = [A]/[N] = x_A/(1 - x_A)$ and the free energy of activation of the enzyme $\Delta G^\circ = -k_\mathrm{B} T \ln K$; with k_B being Boltzmann's constant, T the absolute temperature, and $x_A = [A]/([A] + [N])$ the molar fraction of states A.

ΔG° can be linked to the work that the lateral pressure exerts on the enzymes, by writing the chemical potential for given state $s = A, N$

$$\mu_s = \mu_s^0 + k_\mathrm{B} T \ln x_s - W_s, \tag{4}$$

where μ_s^0 is the standard chemical potential, and W_s is the work associated to the internal lateral pressures[9].

At thermodynamic equilibrium $\mu_A = \mu_N$, from which follows (assuming $\mu_A^0 = \mu_N^0$)

$$\Delta G^{\circ,\text{sym}} - \Delta G^{\circ,\text{asym}} = -\Delta W^{\text{sym}} + \Delta W^{\text{asym}} = -\Delta W. \tag{5}$$

Next, assuming that the total hydrolysis rate depends only on the number of active enzymes, it follows that the ratio of lipid hydrolysis rates in asymmetric and symmetric membranes $r = k_i^{\text{asym}}/k_i^{\text{sym}} \simeq x_A^{\text{asym}}/x_A^{\text{sym}}$, i.e. just depends on the ratio of active proteins in either system. Thus, using Eq. (5), we obtain Eq. (2) in the main text setting $\Delta G^\circ = \Delta G^{\circ,\text{sym}}$.

The work difference between asymmetric and symmetric bilayers is given by [9]

$$\Delta W = -\int dz A(z)\Delta p(z) \approx -\sum_{i=1,2}\sum_{j=\text{out,in}} a_i^j \Delta p_i^j, \tag{6}$$

where z is the transbilayer coordinate, $A(z)$ is the depth-dependent cross-sectional protein area profile, and $\Delta p(z)$ the lateral pressure differences between symmetric and asymmetric bilayers. The approximation of the integral at the right-hand side of the above equation assumes a rotationally symmetric protein shape. Here, $a_i^{\text{out/in}}$ are the coefficients of a Taylor expansion of $A(z)$ in outer and inner leaflets, previously reported for to be $a_1^{\text{out/in}} = 0.344 \pm 0.014$ nm and $a_2^{\text{out/in}} = (2.58 \pm 0.16) \times 10^{-3}$ [6]. $\Delta p_i^{\text{out/in}}$ refers to the difference of the moments of the lateral pressure profiles between symmetric and asymmetric bilayers for each leaflet, where

$$p_1^{\text{out/in}} \simeq \pm\alpha\kappa_m^{\text{out/in}} c_0^{\text{out/in}}, \tag{7}$$

and

$$p_2^{\text{out/in}} = 2h^{\text{out/in}}\alpha\kappa_m^{\text{out/in}} c_0^{\text{out/in}} - \kappa_G^{\text{out/in}}. \tag{8}$$

The correction factor $\alpha = 15.5/14$ accounts for the differential stress induced by the area mismatch in flat asymmetric bilayers[10]; the sign in Equation (7) is negative for the inner leaflet. Further parameters are the monolayer bending rigidity $\kappa_m^{\text{out/in}}$, Gaussian curvature modulus $\kappa_G^{\text{out/in}} \approx -0.8\kappa_m^{\text{out/in}}$ and the position of the neutral plane $h^{\text{out/in}}$. Both moments of the lateral pressure profile were estimated using previously reported structural and elastic data in the absence of ions (see Tab. S1) and via a weighted average according to leaflet composition.

4 Supplementary Tables

Table S1: Membrane structural and elastic parameters used to calculate ΔW. κ_m values were taken from Venable et al.[11]. h values were estimated from structural data (POPC[12], POPE[13], POPG[14]) assuming that the neutral plane coincides with the position of the lipid backbone.

Lipid	c_0 (nm^{-1})	κ_m (k$_B$T)	h (nm)
POPC	0.01±0.04	15.9±0.5	0.96±0.02
POPE	-0.352±0.004	15.6±0.7	1.01±0.02
POPG	0.10±0.04	13.5±0.5	0.96±0.02
POPE (Ca^{2+})	-0.331±0.003	-	-
POPG (Ca^{2+})	-0.20±0.02	-	-
POPE (Na^{+})	-0.333±0.003	-	-
POPG (Na^{+})	-0.15±0.02	-	-

Table S2: Overview of lipid and protein concentrations in all studied samples. Lipid concentrations were determined using a standard phosphate assay[15]. The protein concentration was determined using a Nanodrop ND-1000 spectrophotometer (Peqlab Biotechnology GmbH, Erlangen, Germany) by measuring the absorbance at 280 nm, and using the mass extinction coefficient $\epsilon^{1\%}_{\text{OmpLA}} = 26.68$ Lg^{-1}cm^{-1}.

Sample	system	salt	c_{lipid} (mM)	c_{protein} (μM)
PE/PC$_1$	aPLUVs	Ca^{2+}	1.3	4.6
	sPLUVs	Ca^{2+}	1.5	7.9
PE/PC$_2$	aPLUVs	Ca^{2+}	0.3	1.3
	sPLUVs	Ca^{2+}	1.0	4.6
		Na^{+}	1.4	6.8
PE/PC/PG	aPLUVs	Ca^{2+}	2.6	15.7
		Na^{+}	1.6	9.8
	sPLUVs	Ca^{2+}	2.6	15.7
		Na^{+}	2.1	7.6
PE/PG	aPLUVs	Ca^{2+}	2.9	3.4
		Na^{+}	2.9	3.4
	sPLUVs	Ca^{2+}	2.1	3.3
		Na^{+}	2.1	3.3

References

[1] M. P. K. Frewein, M. Rumetshofer and G. Pabst, *J Appl Crystallogr*, 2019, **52**, 403–414.

[2] A. A. Rieder, D. Koller, K. Lohner and G. Pabst, *Chem Phys Lipids*, 2015, **186**, 39–44.

[3] M. Doktorova, F. A. Heberle, B. Eicher, R. F. Standaert, J. Katsaras, E. London, G. Pabst and D. Marquardt, *Nat Protoc*, 2018, **13**, 2086–2101.

[4] J. Folch, M. Lees, G. H. Sloane Stanley *et al.*, *J Biol Chem*, 1957, **226**, 497–509.

[5] O. L. Knittelfelder and S. D. Kohlwein, *Cold Spring Harb Protoc*, 2017, **2017**, year.

[6] P. Piller, E. F. Semeraro, G. N. Rechberger, S. Keller and G. Pabst, *PNAS Nexus*, 2023, **2**, 1–7.

[7] M. Kaltenegger, J. Kremser, M. P. Frewein, P. Ziherl, D. J. Bonthuis and G. Pabst, *Biochim Biophys Acta*, 2021, **1863**, 183709.

[8] R. M. Neal, *Ann Stat*, 2003, **31**, 705–741.

[9] R. S. Cantor, *J Phys Chem B*, 1997, **101**, 1723–1725.

[10] A. Hossein and M. Deserno, *Biophys J*, 2020, **118**, 624–642.

[11] R. M. Venable, F. L. Brown and R. W. Pastor, *Chemistry and Physics of Lipids*, 2015, **192**, 60–74.

[12] N. Kučerka, M.-P. Nieh and J. Katsaras, *Biochimica et Biophysica Acta (BBA) - Biomembranes*, 2011, **1808**, 2761–2771.

[13] N. Kučerka, B. van Oosten, J. Pan, F. A. Heberle, T. A. Harroun and J. Katsaras, *The Journal of Physical Chemistry B*, 2015, **119**, 1947–1956.

[14] N. Kucerka, B. Holland, J. Pan, F. A. Heberle, C. G. Gray, B. Tomberli and J. Katsaras, *Biophysical Journal*, 2012, **102**, 504a–505a.

[15] G. R. Bartlett, *J Biol Chem*, 1959, **234**, 466–468.
